# Artificial intelligence in automatic image segmentation system for exploring recurrence patterns in small cell carcinoma of the lung

**DOI:** 10.3389/fonc.2025.1534740

**Published:** 2025-05-01

**Authors:** Jing Shen, Shaobin Wang, Hui Guan, Mingyi Di, Zhikai Liu, Qi Chen, Mei Li, Jie Shen, Ke Hu, Fuquan Zhang

**Affiliations:** ^1^ Department of Radiation Oncology, Peking Union Medical College, Beijing, China; ^2^ Product Development Department, MedMind Technology Co, Ltd., Beijing, China; ^3^ Department of Respiratory and Critical Care Medicine, Peking Union Medical College Hospital, Beijing, China

**Keywords:** small cell lung cancer, clinical target volume (CTV), artificial intelligence, local recurrence, prediction model

## Abstract

**Background:**

The integration of artificial intelligence (AI) in automatic image segmentation systems offers a novel approach to evaluating the clinical target volume (CTV) in small cell lung cancer (SCLC) patients. Utilizing imaging recurrence data, this study applies a recursive feature elimination algorithm to model and predict patient prognoses, aiming to enhance clinical guidance and prediction accuracy.

**Materials and Methods:**

This research analyzed data from SCLC patients who received curative radiotherapy from January 1, 2010, to December 30, 2021, and had comprehensive follow-up records including pre- and post-treatment imaging. An AI-driven image segmentation system segmented the initial CTV, evaluating 110 clinical parameters. The recursive feature elimination method selected pertinent features, and a random forest-based recursive prediction model was developed to establish a clinically viable recurrence prediction model.

**Results:**

1. Local Control Analysis: A study of 180 patients, with a median follow-up duration of 36 months, revealed that 94 experienced recurrences, while 86 did not. Factors influencing local control rates included gender (male), age (>60 years), T stage, smoking index, and tumor size. Notably, tumor size (≥ 5cm) emerged as an independent factor significantly impacting local control rates, with a Hazard Ratio (HR) of 1.635 (95% CI: 1.055-2.536, p=0.028). 2. Recurrence Analysis: Tumor size (≥ 5cm) was also closely linked to patient local control rates, with a 3-year Local Control Rate Failure (LCRF) contrasting sharply between larger tumors (61.1%) and smaller tumors (86.7%, p=0.004). Upon analyzing recurrence patterns among 94 patients, a total of 170 instances were examined. Recurrence was most prevalent in regions 10R, 10L, 4R, and 7, accounting for 67.65% (115/170) of cases, while regions 2L and 3P showed no recurrences. The initial region of the primary tumor or metastatic lymph nodes was identified as a critical recurrence area, with a 100% recurrence rate in patients whose initial tumor region included 10 specific regions. The recurrence rates for initial tumor regions involving 4R, 7, 11R, and 11L ranged between 41.6% and 45.5%. 3. Development of a recurrence prediction model: utilizing an AI-powered automatic image segmentation system, multidimensional partition parameters, including 110 clinical variables, were analyzed. The recursive feature elimination method facilitated efficient feature selection. From this, a recurrence prediction model for small cell lung cancer was developed using a random forest algorithm, achieving a clinically significant accuracy rate of 77%. This model provides a reliable basis for enhancing the clinical application and decision-making process for medical practitioners.

**Conclusion:**

The utilization of AI-based automatic image segmentation system for delineating the initial CTV has proven pivotal. Analysis and modeling of recurrent images reveal that the initial GTV and GTVnd are critical regions for recurrence. Leveraging partition parameter and variable information, we constructed a clinically viable recurrence prediction model. This model significantly aids in guiding the precise clinical targeting of treatment areas, demonstrating the potential of AI to enhance patient management and treatment outcomes in small cell lung cancer.

## 1 Introduction

Lung cancer is the second most common tumor worldwide and accounts for approximately 18.0% of cancer-related mortality (1). Treatment of lung cancer includes a combination of surgery, radiotherapy, and systemic therapy (chemotherapy, immunotherapy, and targeted agents). Among these therapeutic modalities, radiotherapy is the only one that is indicated at all stages of the disease, in all pathologic types and physical states (2, 3).

The local recurrence rate of small-cell lung cancer is still high and seriously affects the survival of patients, especially recurrence within the radiotherapy field. Related studies on the prediction of recurrence models are mostly in clinical and imaging indexes, etc., and the models for evaluation and prediction are less reproducible and less accurate.

In recent years, with the great development of computer application in radiotherapy (RT), we can collect medical image data, clinical information, treatment records, etc. of patients with small cell lung cancer. Image segmentation is performed using deep learning techniques, such as convolutional neural network (CNN) (4–7). Train a network to automatically mark tumor tissue and normal tissue in the image. Features related to recurrence are extracted from image data. This may include the size, shape, location and other information of the tumor. These characteristics will help to analyze patterns associated with recurrence. Recursive feature elimination (RFE) algorithm is used to select features that are important for predicting recurrence, so as to improve the generalization ability of the model and help reduce over fitting. Through cross validation and other methods to evaluate the performance of the model, optimize the model parameters, and predict the possibility of its recurrence. This can provide doctors with auxiliary information to help them make more accurate clinical decisions.

Our previous research has confirmed that it can be applied to the delineation of clinical target volume (CTV) and organ at risk (OAR) of lung cancer, as well as to the application of artificial intelligence in automatic image segmentation system (8). Based on this, our research aims to evaluate, analyze, and model clinical targets in different lymphatic drainage areas based on artificial intelligence, It can also predict the prognosis and play a role in clinical guidance and prediction.

## 2 Materials and methods

### 2.1 Patients’ data and information

**Figure 1:**
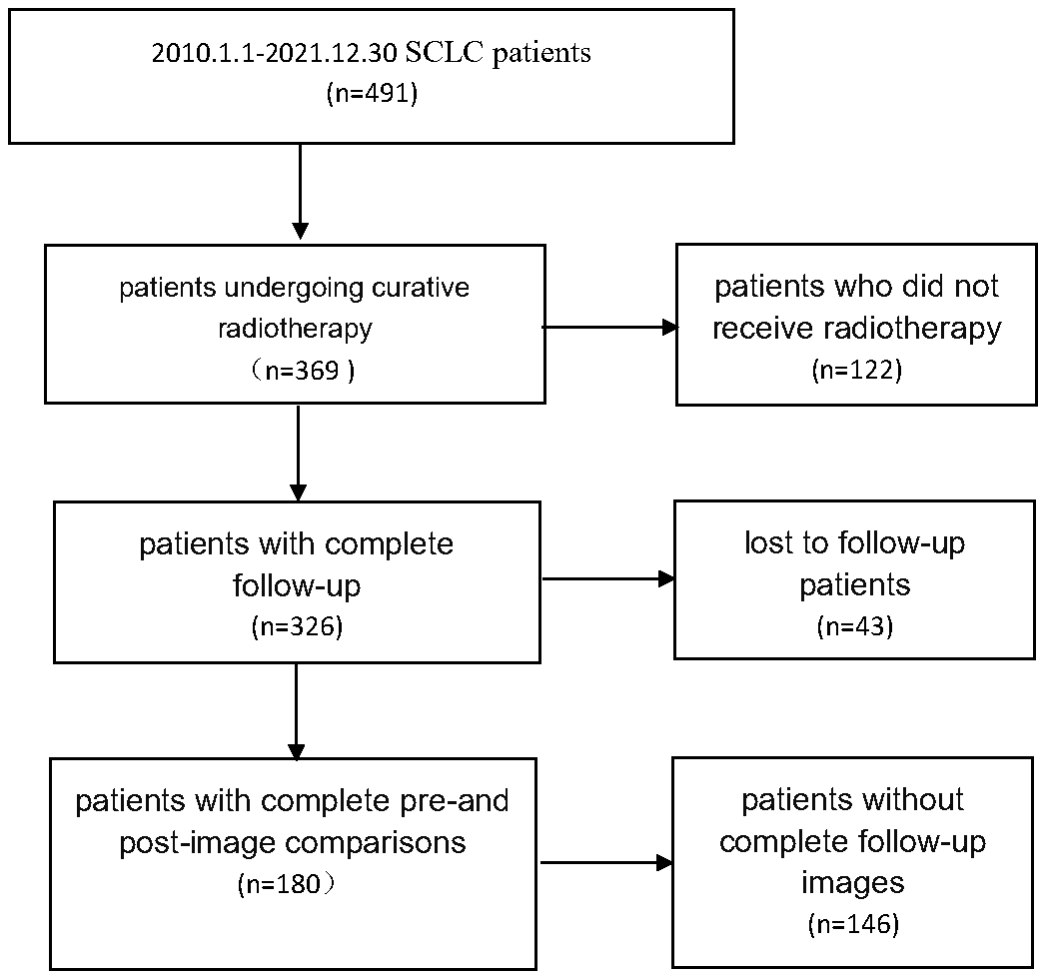
Patient screening flowchart.

Small cell lung cancer patients from 2010.1.1 to 2021.12.30 were collected, and 180 patients who underwent radical radiotherapy with complete follow-up records and complete before and after image comparisons were collected. The details are shown in **Figure 1**.

A total of 180 patients were enrolled in the group, all of whom had complete imaging evaluation before treatment, compared images of the location of the recurrent focus, collected 16676 CT slices for localization, delineated the mediastinum, hilar lymph nodes and GTVnd layer by layer, and corrected the delineated CTV. All patients were scanned with Philips Brilliance Big Bore CT scanner before receiving radiotherapy, and the protocol of digital imaging and communications in medicine (DICOM) was followed. The matrix size of CT image is 512 × 512, the pixel spacing is 1.1543mm × 1.1543mm, and the layer thickness is 5mm. Keep patient information confidential during data collection and processing.

### 2.2 Perform regional segmentation of the mediastinal lymphatic drainage area

This study divides hilar and mediastinal lymph nodes into 14 stations according to the definition of boundary of mediastinal lymph node division by the International Association for the Study of Lung Cancer (IASLC) and the definition of lymph node division by the UICC and AJCC. The specific partitions are shown in **Appendix 1**. The clinical positioning CT images of 180 patients are divided into regions.

### 2.3 Follow-up recurrence information, search for predictive models

For the recurrence pattern and specific recurrence location of follow-up patients, the initial CTV of patients is divided into regions, and a recurrence prediction model based on partition information is established.

#### 2.3.1 Automatic partition of CTV based on artificial intelligence

**Figure 2:**
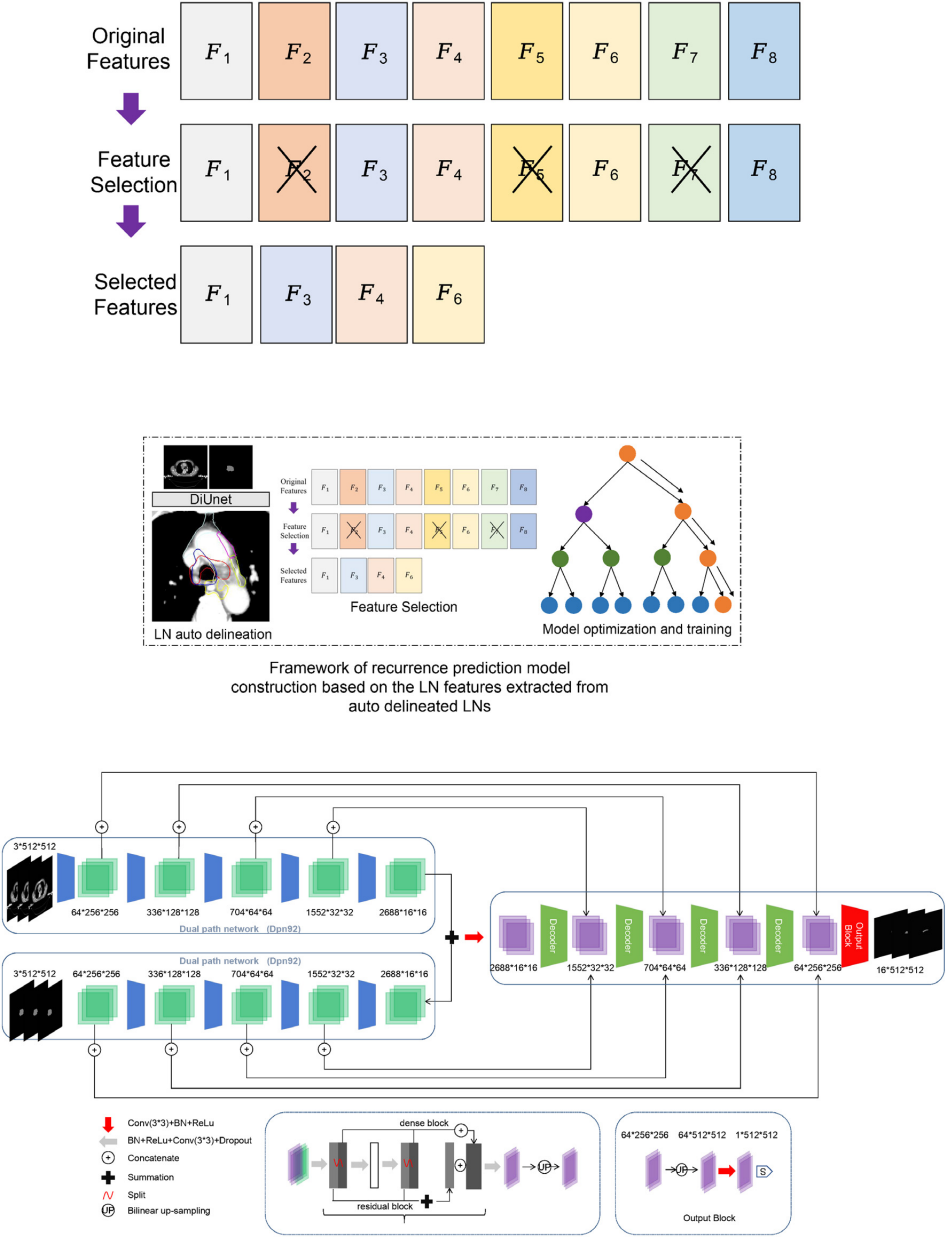
Framework of recurrence prediction model construction based on the LN features extracted from auto delineated LNs. **(A)** recurrence prediction model construction, **(B)** Framework of DiUnet.

The previously published DiUnet (see **Figure 2**) is used for automatic CTV partitioning. This model uses the region of GTV as prior knowledge to fuse into the U-net model, uses GTV and CT images as dual channels for coding, extracts the image features of the two channels, and then carries out feature fusion to improve the accuracy of boundary recognition near GTV.

#### 2.3.2 Clinical parameter/variable information

After the 14 station area is automatically divided (See **Table 1**), the partition parameters can be calculated, and the ideaCTV is defined as the union area of all partitions violated by the CTV,


$$ideaCTV=LN_{m}\cup \;LN_{n},\;m,n\in \left[1,\;16\right]and\left|CTV\cap \;LN_{m}\right|>0and\left|CTV\cap \;LN_{n}\right|>0$$

**Table 1:** Correspondence between LN and partition.

$LN_{1}$	$LN_{2}$	$LN_{3}$	$LN_{4}$	$LN_{5}$	$LN_{6}$	$LN_{7}$	$LN_{8}$	$LN_{9}$	$LN_{10}$	$LN_{11}$	$LN_{12}$	$LN_{13}$	$LN_{14}$	$LN_{15}$	$LN_{16}$
1R	1L	2R	2L	3A	3P	4R	4L	5	6	7	8	10R	10L	11R	11L

The formula for each partition parameter is:

Volume ratio of CTV in LN


$${\text{RctvLN}}_{i}=\frac{\left|CTV\cap \;LN_{i}\right|}{\left|LN_{i}\right|},i\in \left[1,\;16\right]$$

CTV volume ratio in ideaCTV


$${\text{RIctvLN}}_{i}=\frac{\left|CTV\cap \;LN_{i}\right|}{\left|ideaCTV\right|},\;i\in \left[1,\;16\right]$$

Volume ratio of GTV in LN


$${\text{RgtvLN}}_{i}=\frac{\left|GTV\cap \;LN_{i}\right|}{\left|LN_{i}\right|},\;i\in \left[1,\;16\right]$$

Volume proportion of GTV in ideaCTV


$${\text{RIgtvLN}}_{i}=\frac{\left|GTV\cap \;LN_{i}\right|}{\left|ideaCTV\right|},\;i\in \left[1,\;16\right]$$

Volume ratio of CTV-GTV in LN


$${\text{RcmgLN}}_{i}=\frac{\left|\left(CTV-GTV\right)\cap \;LN_{i}\right|}{\left|LN_{i}\right|},\;i\in \left[1,\;16\right]$$

Volume ratio of CTV GTV in ideaCTV


$${\text{RIcmgLN}}_{i}=\frac{\left|\left(CTV-GTV\right)\cap \;LN_{i}\right|}{\left|ideaCTV\right|},\text{ i}\in \left[1,\;16\right]$$

There are 110 partition parameter/variable information (See **Table 2**), including voxel resolution, CTV image volume, CTV volume proportion in LN, GTV size before radiotherapy, GTV image volume, GTV volume proportion in LN, lung area location and lymph drainage area coverage.

**Table 2:** Parameter information.

Number	Parameter	Parameter interpretation	Assignment
1-16	${\text{RctvLN}}_{i}$	Volume ratio of CTV in LN	0.00-1.00
17-32	${\text{RIctvLN}}_{i}$	CTV volume ratio in ideaCTV	0.00-1.00
33-48	${\text{RgtvLN}}_{i}$	Volume ratio of GTV in LN	0.00-1.00
49-64	${\text{RIgtvLN}}_{i}$	Volume proportion of GTV in ideaCTV	0.00-1.00
65-80	${\text{RcmgLN}}_{i}$	Volume ratio of CTV-GTV in LN	0.00-1.00
81-96	${\text{RIcmgLN}}_{i}$	Volume ratio of CTV GTV in ideaCTV	0.00-1.00
97		CTV volume	6412-165390
98		GTVnd volume	0-76514
99		Image resolution	0.89-1.37
100		The number of LNs involved in CTV	0-16
101		The number of LNs involved in GTVnd	0-16
102		Location of GTV in the lung area	1,2,3,4
103-109		Any overlap between 4L, 4R, 5,6,7,11L, and 11R and CTV	0 or 1
110		GTV size before radiotherapy cm^^3^	1.31-11.72

#### 2.3.3 Model building: standardize the extracted feature parameters and reduce the dimension of features

Firstly, the correlation coefficient between each feature and other features is calculated, and redundant features are removed according to the correlation coefficient; To mitigate overfitting, the dataset was divided into training (80%) and validation (20%) subsets. The recursive feature elimination (RFE) process incorporated 5-fold cross-validation during feature selection to prioritize generalizable features. The random forest algorithm’s inherent bagging mechanism further reduces overfitting by aggregating predictions from multiple decision trees. Hyperparameters, including tree depth and the number of estimators, were optimized using grid search to balance model complexity and performance. Then, a new feature set is constructed with the remaining features, and the next round of training is carried out until all features are traversed. After feature screening, we reserved 27 feature dimensions.

The random forest method was used to build a prediction model for lung cancer recurrence (9). Random Forest Classifier (RF) is an algorithm that integrates multiple trees using integrated learning theory. Its basic unit is the decision tree. After random sampling of the original training data, use each decision tree in the forest to judge the unlabeled samples, and then apply the majority voting results of all decision trees to predict the unlabeled sample categories. This method has a relatively low trend of over fitting, and the model is as follows:


$$RF\left(x\right)=\frac{1}{B}\sum_{i=1}^{B}T_{i,z_{i}}\left(x\right)$$

Among them, RF(x) denotes the random forest pair sample x . The predicted B  value of the Indicates that there are a total of B Tree. $z_{i\;}$ Indicates that the first the i training set used for the tree, the $T_{i}$ Indicates that the first i tree learner.

## 3 Results

### 3.1 Basic patient information

180 patients, the median age is 60 years old, 133 male patients (73.8%), 47 female patients (26.1%), 3 patients (1.7%) with complex pathological type, respectively, large cell neuroendocrine carcinoma, atypical carcinoid, adenocarcinoma, 142 central type tumors (78.9%), 38 peripheral type tumors (21.1%), 155 patients (86.1%), 28 patients with paraneoplastic syndrome, Including 12 cases of SIADH, 3 cases of LAMBERT, 5 cases of ectopic ACTH, 8 cases of anti GABA2 receptor encephalitis, 119 cases (66.1%) of patients received synchronous radiotherapy and chemotherapy, 6MV X-ray radiotherapy, intensity modulated radiotherapy, 60Gy/30f, 2Gy/f, conventional segmentation mode, see **Table 3** for details.

**Table 3:** Basic clinical information of the patients.

Characteristics	n	%
Age		
Median	60	
Average	62	
Gender		
Male	130	72.2
Female	50	27.8
Pathological type		
Small cell only	176	97.8
Small cell mixed type	4	2.2
Tumor location		
Central type	136	75.6
Peripheral type	44	24.4
T stage		
T1	28	15.6
T2	47	26.1
T3	21	11.7
T4	84	46.7
N Stage		
N0	6	3.3
N1	8	4.4
N2	82	45.6
N3	84	46.7
TNM Stage		
I	4	2.2
II	4	2.2
III	158	87.8
IV	14	7.8
Paraneoplastic syndrome		
Yes	26	14.4
No	154	85.6
Superior vena cava syndrome		
Yes	12	6.7
No	168	93.3
Smoking index		
0	59	32.8
0-400	12	6.7
>400	109	60.5
Chemotherapy		
Synchronous radiochemotherapy	119	66.1
Sequential radiochemotherapy	61	33.9

### 3.2 Summary of recurrence

180 patients, median follow-up time 36 months, 94 patients with recurrence, 86 patients without recurrence, statistical clinical indicators include: whether the age is over 60, gender, whether other pathological types are mixed, tumor location (central type, peripheral type), T stage, N stage, clinical stage, whether there is paraneoplastic syndrome, smoking index The results showed that gender, age over 60, T stage, smoking index, and tumor size were related to the local control rate of patients. The tumor size was an independent factor related to the local control rate, HR 1.635 (95% CI 1.055-2.536), p=0.028. See **Table 4** for details.

**Table 4:** Univariate and multivariate analyses related to localized control rates.

Characteristics		Univariate analyses				Multivariate analyses		
		N	3-year LC		P value	HR	95% CI	P value
			No relapse	Relapse				
Gender	Male	130	48	82	0.000			
	Female	50	38	12	*			
Age	<60	82	46	36	0.001			
	≥60	98	40	58	*			
Pathological type	Small cell	176	84	92	0.477			
	Small cell mixed type	4	2	2				
Tumor location	Central type	136	59	77	0.102			
	Peripheral type	44	27	17				
T stage	T1	28	22	6	0.019			
	T2	47	15	32	*			
	T3	21	10	11				
	T4	84	39	45				
N stage	N0	6	4	2	0.622			
	N1	8	2	6				
	N2	82	43	39				
	N3	84	37	47				
TNM stage	I	4	2	2	0.802			
	II	4	2	2				
	III	158	75	83				
	IV	14	7	7				
Paraneoplastic syndrome	Yes	26	13	13	0.104			
	No	154	73	81				
superior vena cava syndrome	Yes	12	6	6	0.235			
	No	168	106	62				
smoking index	0	59	45	14	0.001*			
	0-400	12	4	8				
	>400	109	35	74				
Tumor diameter size	<5cm	96	65	31	0.004*	1.635	1.055-2.536	0.028*
	≥5cm	84	21	63				
Chemotherapy	Synchronous radiochemotherapy	119	50	69				
	Sequential radiochemotherapy	61	25	36				

#### 3.2.1 Relationship between tumor size and recurrence

**Figure 3:**
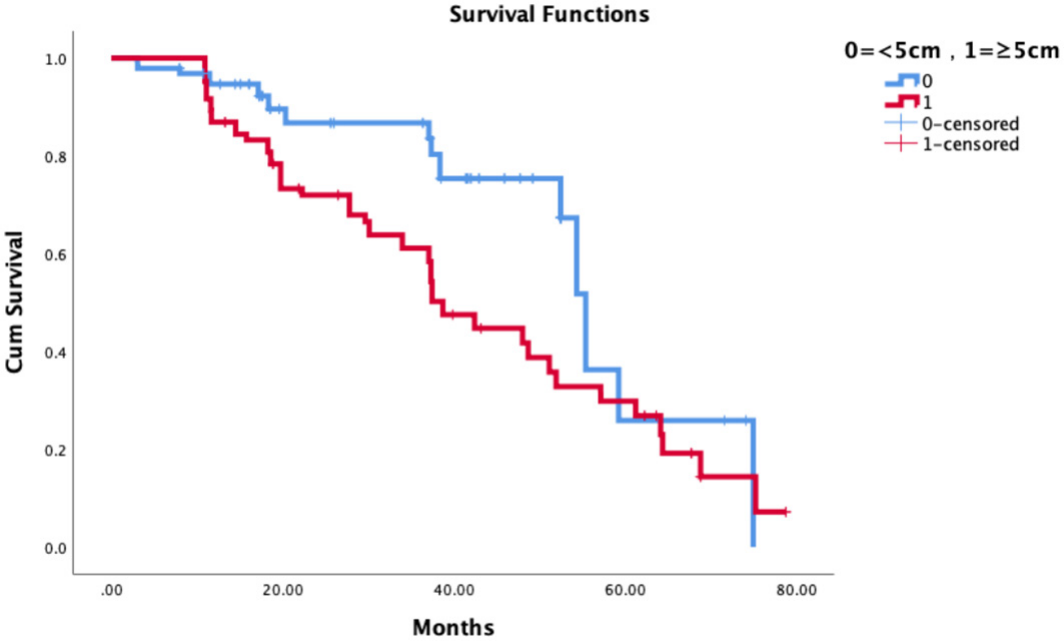
Graph of tumor size in relation to local recurrence.

The median follow-up was 36 months. The tumor size was related to the local control rate of patients and was an independent correlation factor of the local control rate. The 3-year LCRF was 86.7% vs 61.1%, p=0.004. See **Figure 3** for details.

#### 3.2.2 Tumor location in relation to recurrence

Further, 94 patients with recurrence were divided according to regions, and a total of 170 cases were counted. The recurrence in 10R, 10L, 4R and 7 areas is the main, accounting for 67.65% (115/170), including 2 cases in 1L area, 3 cases in 1R area, 8 cases in 2R area, 3 cases in 3A area, 3 cases in 4L area, 24 cases in 4R area, 3 cases in 5 areas, 10 cases in 6 areas, 18 cases in 7 areas, 2 cases in 8 areas, 23 cases in 10L area, 50 cases in 10R area, 10 cases in 11L area, 11 cases in 11R area, and no recurrence in 2L area and 3P area. See **Table 5** for details.

**Table 5:** Recurrence statistics in different lymphatic drainage zones.

Area	Number of regional recurrence	Actual delineation of recurrence area/proportion of idea area (Mean)	Actual delineation of recurrence area/proportion of idea area(range)	GTV affected area	CTV-GTV recurrence cases
1R	3	0.426	0-0.85	0	3
1L	2	0	0	0	2
2R	8	0.197	0-0.70	0	8
2L	0	–	–	0	0
3A	3	0.030	0.01-0.09	0	3
3P	0	–	–	0	0
4R	24	0.476	0-0.94	10	14
4L	3	0.463	0-0.98	2	1
5	3	0.908	0.73-1	2	1
6	10	0.114	0-0.57	1	9
7	18	0.559	0-0.90	8	10
8	2	0.551	0-0.87	0	2
10R	50	0.715	0-1	50	0
10L	23	0.651	0-1	23	0
11R	11	0.593	0-1	5	6
11L	10	0.641	0.15-0.82	4	6
Total	170				

The initial area of the primary focus or metastatic lymph node is the key area of recurrence, mainly 10R and 10L. Patients with the initial tumor area involving 10 areas all experienced recurrence (100%). Patients with the initial tumor area involving 4R, 7, 11R and 11L also had a certain recurrence rate, 41.6% - 45.5%. See Appendix 2 for details.

The automatic image segmentation system of human intelligence is applied to carry out partition statistics on the initial clinical CTV of patients, and calculate the proportion range of the actual sketched/standard area of the recurrence area. The results show that,

### 3.3 Recurrence pattern searching

According to the multidimensional parameters in the machine learning prediction model, including 110 clinical parameters/variables information, the recurrence prediction model of small cell carcinoma of the lung was constructed based on the random forest machine learning method, and the exploration of the establishment of a clinically usable recurrence prediction model is detailed in **Table 6**.

**Table 6:** Relevant screening characteristics of the relapse prediction model.

Number	Parameter	Parameter interpretation
2	${\text{RctvLN}}_{2}$	The proportion of CTV in $LN_{2}$ volume
5	${\text{RctvLN}}_{5}$	The proportion of CTV in $LN_{5}$ volume
6	${\text{RctvLN}}_{6}$	The proportion of CTV in $LN_{6}$ volume
7	${\text{RctvLN}}_{7}$	The proportion of CTV in $LN_{7}$ volume
8	${\text{RctvLN}}_{8}$	The proportion of CTV in $LN_{8}$ volume
9	${\text{RctvLN}}_{9}$	The proportion of CTV in $LN_{9}$ volume
18	${\text{RIctvLN}}_{2}$	The volume proportion of CTV in the overlapping area of CTV and $LN_{2}$ in ideaCTV
23	${\text{RIctvLN}}_{7}$	The volume proportion of CTV in the overlapping area of CTV and $LN_{7}$ in ideaCTV
24	${\text{RIctvLN}}_{8}$	The volume proportion of CTV in the overlapping area of CTV and $LN_{8}$ in ideaCTV
30	${\text{RIctvLN}}_{14}$	The volume proportion of CTV in the overlapping area of CTV and $LN_{14}$ in ideaCTV
34	${\text{RgtvLN}}_{2}$	The proportion of GTV in $LN_{2}$ volume
40	${\text{RgtvLN}}_{8}$	The proportion of GTV in $LN_{8}$ volume
41	${\text{RgtvLN}}_{9}$	The proportion of GTV in $LN_{9}$ volume
43	${\text{RgtvLN}}_{11}$	The proportion of GTV in $LN_{11}$ volume
46	${\text{RgtvLN}}_{14}$	The proportion of GTV in $LN_{14}$ volume
55	${\text{RIgtvLN}}_{7}$	The volume proportion of GTV in the overlapping area of GTV and $LN_{7}$ in ideaCTV
58	${\text{RIgtvLN}}_{10}$	The volume proportion of GTV in the overlapping area of GTV and $LN_{10}$ in ideaCTV
61	${\text{RIgtvLN}}_{13}$	The volume proportion of GTV in the overlapping area of GTV and $LN_{13}$ in ideaCTV
98		GTVnd volume
99		Image resolution
101		The number of LNs involved in GTVnd
110		GTV size before radiotherapy cm^^3^

**Figure 4:**
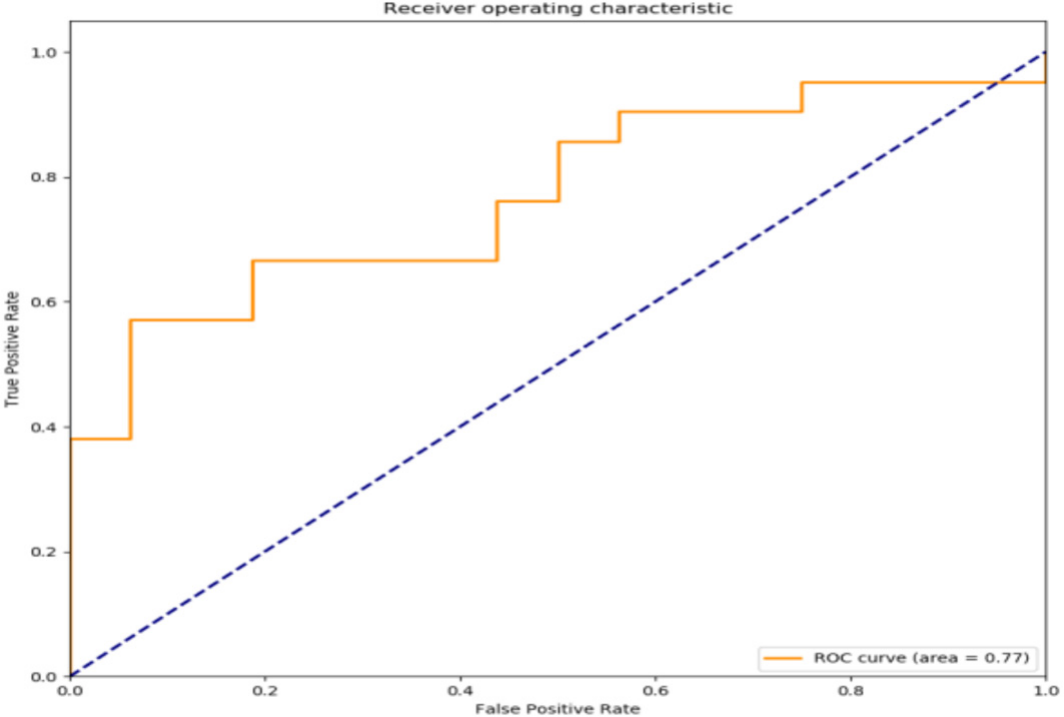
ROC curve of prediction model.

The filtered characteristics are shown in the following table. The ROC curve of the model built based on the filtered features is shown in **Figure 4**. The AUC value of this model is 0.77 in **Table 7**. The DiUnet segmentation achieved high accuracy across all lymph node regions, with DSC >0.85 for stations 4R, 7, and 10R, which are critical for recurrence prediction. Lower DSC (0.78) was observed in smaller regions (e.g., 3P), likely due to limited anatomical visibility on CT.

**Table 7:** Performance metrics of the random forest model on training and validation sets for SCLC recurrence prediction.

Metric	Training Set	Validation Set
Accuracy	82%	77%
AUC	0.85	0.77
Precision	0.81	0.75
Recall	0.84	0.73
F1-Score	0.82	0.74

## 4 Discussion

Lung cancer usually involves multiple treatments, and 77% of lung cancer patients have evidence-based indications for radiotherapy (10–13). The use of radiotherapy can improve the local control rate and overall survival rate of lung cancer (14–16). The success of radiotherapy depends on the accurate irradiation of tumor targets. How to accurately delineate the clinical target volume (CTV) is a crucial step for the best effect of radiotherapy (17, 18). In clinical work, the quality of manual delineation of clinical target areas depends on the prior knowledge and clinical experience of radiation oncologists. However, images drawn by different doctors or the same doctor at different times will be different. However, clinical target delineation based on automatic segmentation technology of artificial intelligence can provide efficient and accurate delineation results, which have been confirmed in our previous research (8, 19). Based on our previous research, we can explore the integrity and accuracy of the involved lymph node region, and its impact on survival prognosis, local recurrence rate and lymph node failure rate. Based on this, our research uses the automatic image segmentation system of artificial intelligence to segment the clinical initial CTV region, analyze the recurrence image, and establish a clinically available recurrence prediction model using a random forest classifier, which plays a certain role in guiding the clinical application of actual CTV delineation.

At present, the research on clinical targets based on artificial intelligence and the method based on convolutional neural network (CNN) have been successfully applied to the delineation of dangerous organs and clinical targets of lung cancer. The published data related to deep learning mainly focus on the segmentation of postoperative radiotherapy (PORT) (19), dangerous organs (OAR) (20–23), and primary lung tumors (24), but there is no relevant research to delineate and evaluate the mediastinal lymph node region. Based on this, this study uses an automatic image segmentation system of human intelligence to perform the partition statistics of patients’ initial clinical CTV, The application includes clinical parameter/variable information, and recursive feature elimination method is used to select other features. A recursive prediction model with random forest is constructed by using the selected features.

In this study, 94 patients with recurrence among 180 patients were followed up for a median of 36 months. AI-driven automatic image segmentation system was used to partition the initial clinical CTV of patients. The results showed that patients in the area where the initial tumor was located involved in 10 areas had recurrence, and patients in the area where the initial tumor was located involved in 4R, 7, 11R, 11L also had a recurrence rate of 41.6% - 45.5%, The area where the primary focus or metastatic lymph node is initially located is the key area of recurrence. Therefore, the application includes 110 clinical parameter/variable information, and the recursive feature elimination method is used to select other features. The selected features are used to build a lung cancer recurrence prediction model based on the random forest classifier. The model demonstrates robust clinical feasibility for recurrence prediction.

This study is the first one to apply a human-intelligent automatic image segmentation system to accurately define and automatically segment the boundaries of mediastinal and hilar lymph node regions for clinical recurrence prediction modeling in radiation therapy for lung cancer. This study can provide valuable guidance for clinicians. This includes the possibility of integrating predictive modeling with actual clinical decision making. This may require more validation and practice, but it is expected to provide a more personalized treatment approach in the future.

This study not only demonstrates the clinical value of artificial intelligence (AI) in contouring the clinical target volume (CTV) and analyzing recurrence patterns in small cell lung cancer, but also provides an innovative teaching tool for medical education. The automatic segmentation model and recurrence prediction model based on DiUNet can serve as a teaching platform that combines theory and practice. This helps young doctors quickly master image segmentation techniques and recurrence pattern analysis, shortening the learning curve. By using the AI system, students can adjust and optimize segmentation results in practical operations. They can also perform case analyses using recurrence case data to develop clinical thinking skills. Moreover, the multi-disciplinary team cooperation model offers opportunities for collaborative learning in education. In the future, this model is expected to be extended to a broader field of medical education. It will provide support for training medical professionals with innovative and practical skills. There are some limitations in this study, firstly, this study is a single-center data with a small data sample size. This possible bias in the process of data collection and analysis can be followed by further expansion of the validation of larger samples, integration with data from other medical centers, and improvement of prediction models. In addition, observer bias from oncologist evaluation was unavoidable in this study. We believe that this method should be extended to other thoracic cancers involving the mediastinal lymphatic drainage region, such as esophageal cancer.

The possible aspects of patient privacy, data use and safety in this study were reviewed by the Ethics Committee of Peking Union Medical College Hospital.

The model’s AUC of 0.77 on the validation set underscores its generalizability, though future multi-center studies are needed to confirm robustness. The inclusion of segmentation metrics (DSC, HD) ensures the reliability of feature extraction, a cornerstone for predictive accuracy in AI-driven oncology models.

## 5 Conclusion

The automatic image segmentation system of artificial intelligence is used to segment the initial CTV of small cell lung cancer patients. Based on the analysis and modeling of recurrent images, the results show that the initial tumor GTV and the initial metastatic lymph node GTVnd are the key areas of recurrence. According to clinical parameters/variable information, the use of random forest classifier can establish a clinically available recurrence prediction model, it plays a guiding role in the clinical application of CTV delineation, and has certain clinical practicability.

## Data Availability

The original contributions presented in the study are included in the article/supplementary material. Further inquiries can be directed to the corresponding author.

## Appendix 1 14-station mediastinal lymph node compartmentalization definitions.

**Table:**

7 Zone	14 stations
Supraclavicular region	1R: Right lower neck lymph nodes, supraclavicular lymph nodes, sternal notch lymph nodes 1L: Left lower neck lymph nodes, supraclavicular lymph nodes, sternal notch lymph nodes
Upper region Upper mediastinal lymph nodes	2R: Right upper paratracheal lymph node 2L: Left upper paratracheal lymph node 3A: Prevascular lymph nodes 3P: Retrotracheal lymph nodes 4R: Right lower tracheal paralymph node 4L: Left lower tracheal paratracheal lymph node
Main pulmonary artery area	5: Subaortic lymph nodes 6: Paraaortic lymph nodes
Subprotuberance zone	7: Subprotuberance lymph nodes
Lower zone Lower mediastinal lymph nodes	8: Paraesophageal lymph nodes 9: Pulmonary ligament lymph nodes
Hilar and interlobular regions of the lungs Pulmonary lymph nodes	10: Hilar lymph nodes 11: Interlobular lymph nodes
